# A Multi-component Intervention (NEXpro) Reduces Neck Pain-Related Work Productivity Loss: A Randomized Controlled Trial Among Swiss Office Workers

**DOI:** 10.1007/s10926-022-10069-0

**Published:** 2022-09-27

**Authors:** Andrea Martina Aegerter, Manja Deforth, Thomas Volken, Venerina Johnston, Hannu Luomajoki, Holger Dressel, Julia Dratva, Markus Josef Ernst, Oliver Distler, Beatrice Brunner, Gisela Sjøgaard, Markus Melloh, Achim Elfering, the NEXpro Collaboration Group, Andrea Martina Aegerter, Manja Deforth, Thomas Volken, Venerina Johnston, Hannu Luomajoki, Holger Dressel, Julia Dratva, Markus Josef Ernst, Oliver Distler, Beatrice Brunner, Gisela Sjøgaard, Markus Melloh, Achim Elfering

**Affiliations:** 1 Institute of Public Health, School of Health Sciences, ZHAW Zurich University of Applied Sciences, Katharina Sulzer-Platz 9, 8400 Winterthur, Switzerland; 2 Epidemiology, Biostatistics and Prevention Institute, Department of Biostatistics, University of Zurich, Zurich, Switzerland; 3 School of Health and Rehabilitation Sciences, The University of Queensland, Brisbane, QLD Australia; 4 Institute of Physiotherapy, School of Health Sciences, ZHAW Zurich University of Applied Sciences, Winterthur, Switzerland; 5 Division of Occupational and Environmental Medicine, Epidemiology, Biostatistics and Prevention Institute, University Hospital Zurich, University of Zurich, Zurich, Switzerland; 6 Faculty of Medicine, University of Basel, Basel, Switzerland; 7 Centre of Precision Rehabilitation for Spinal Pain, School of Sport, Exercise & Rehabilitation Sciences, University of Birmingham, Birmingham, UK; 8 Department of Rheumatology, University Hospital Zurich, University of Zurich, Zurich, Switzerland; 9 Winterthur Institute of Health Economics, School of Management and Law, ZHAW Zurich University of Applied Sciences, Winterthur, Switzerland; 10 Department of Sports Science and Clinical Biomechanics, University of Southern Denmark, Odense, Denmark; 11 Faculty of Health, Victoria University of Wellington – Te Herenga Waka, Wellington, New Zealand; 12 Curtin Medical School, Curtin University, Bentley, WA Australia; 13 School of Medicine, The University of Western Australia, Perth, WA Australia; 14 Institute of Psychology, University of Bern, Bern, Switzerland

**Keywords:** Absenteeism, Ergonomics, Exercise, Health promotion, Presenteeism

## Abstract

*Purpose * Neck pain is common among office workers and leads to work productivity loss. This study aimed to investigate the effect of a multi-component intervention on neck pain-related work productivity loss among Swiss office workers. *Methods* Office workers, aged 18–65 years, and without serious neck-related health problems were recruited from two organisations for our stepped-wedge cluster randomized controlled trial. The 12-week multi-component intervention included neck exercises, health-promotion information, and workplace ergonomics. The primary outcome of neck pain-related work productivity loss was measured using the Work Productivity and Activity Impairment Questionnaire and expressed as percentages of working time. In addition, we reported the weekly monetary value of neck pain-related work productivity loss. Data was analysed on an intention-to-treat basis using a generalized linear mixed-effects model. *Results* Data from 120 participants were analysed with 517 observations. At baseline, the mean age was 43.7 years (SD 9.8 years), 71.7% of participants were female (*N* = 86), about 80% (*N* = 95) reported mild to moderate neck pain, and neck pain-related work productivity loss was 12% of working time (absenteeism: 1.2%, presenteeism: 10.8%). We found an effect of our multi-component intervention on neck pain-related work productivity loss, with a marginal predicted mean reduction of 2.8 percentage points (*b* = −0.27; 95% CI: −0.54 to −0.001, *p* = 0.049). Weekly saved costs were Swiss Francs 27.40 per participant. *Conclusions:* Our study provides evidence for the effectiveness of a multi-component intervention to reduce neck pain-related work productivity loss with implications for employers, employees, and policy makers.

*Trial Registration* ClinicalTrials.gov, NCT04169646. Registered 15 November 2019—Retrospectively registered, https://clinicaltrials.gov/ct2/show/NCT04169646.

**Supplementary Information:**

The online version contains supplementary material available at 10.1007/s10926-022-10069-0.

## Introduction

Non-specific neck pain is one of the most common musculoskeletal disorders worldwide and ranked fourth in terms of disability in the 21st century [1]. The 12-month-prevalence of neck pain ranges from 30 to 50% [2], with recurrence rates of 50–75% within the first 5 years of onset [3]. Especially among office workers, neck pain is one of the most frequently reported complaints: About 68% of Swiss office workers experience at least 1 day per year with non-specific neck pain [4], and one in four report work productivity loss due to neck/shoulder pain [5].

Neck pain imposes an impact at the individual and societal level. At the individual level, there is reduced function and quality of life, increased pain and disability [1]. At a societal level, neck pain has health-related economic consequences [1, 2]. In Switzerland, for example, the annual direct costs of neck and back pain amount to Swiss Francs (CHF) 3.8 billion, and the indirect costs, including absenteeism and presenteeism, to CHF 7.5 billion [6]. These consequences become more relevant considering neck pain has a high recurrence rate (e.g., flare-ups) and risk of persistence [1, 2]. Thus, the need to minimize the burden of neck pain among office workers is of interest to many, not only the affected persons themselves, but also the employers and insurance companies.

From an employer’s perspective, current literature describes various approaches to reducing neck pain-related productivity loss in the workplace. Two studies found a positive effect of workplace health promotion alone on health-related work productivity, absenteeism, and presenteeism [7, 8]. Workstation ergonomics alone was shown to positively influence productivity in asymptomatic office workers [9] and absenteeism in office workers with upper limb symptoms [10], but not in office workers with neck pain [11]. Workplace-based exercise was able to reduce neck pain among office workers [12, 13] with work productivity and absenteeism remaining unchanged [11, 14]. Interestingly, several studies on workplace strengthening exercises concluded that exercise frequency was not related to a reduction of neck pain [15, 16]. Pereira and colleagues [17] studied a combination of the previously mentioned intervention approaches and showed that office workers with neck pain who attended a best practice workplace ergonomics and neck exercise programme had a lower absenteeism than those who attended a workplace ergonomics and health promotion programme. In summary, the different approaches—whether applied as a single or combined intervention—provide mixed findings, mostly with small effects, on work productivity loss among symptomatic and asymptomatic office workers. However, the neck pain-related productivity improvements among office workers may be greater if available and best-evidence interventions were combined and tested against a true control group [17–19].

The aim of this trial was therefore to investigate the effect of a multi-component intervention on neck pain-related work productivity loss in office workers. We hypothesised that our multi-component intervention would reduce the economic burden of neck pain in office workers by improving neck pain-related work productivity.

## Methods

### Study Design

**Fig. 1:**
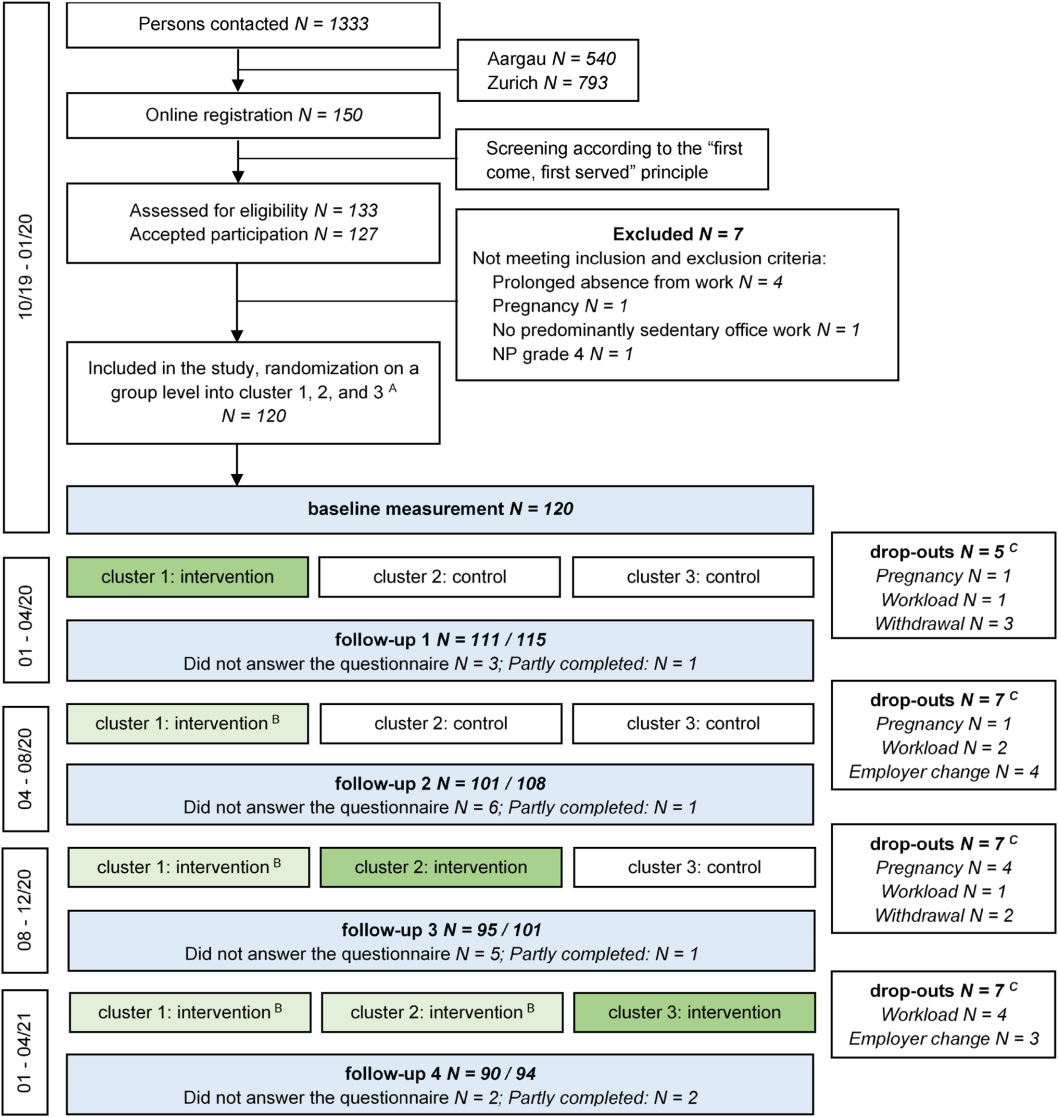
Trial profile. ^A^: each cluster consists of five groups with eight participants each (*N* = 40), cluster 1: two groups from Aargau (*N* = 16) and three groups from Zurich (*N* = 24), cluster 2: one group from Aargau (*N* = 8) and 4 groups from Zurich (*N* = 32), cluster 3: four groups from Aargau and one group from Zurich (*N* = 8) ^B^: unsupervised intervention. ^C^: *N* = 107 participants started the intervention at the allocated time point, *N* = 7 dropped out during the (supervised) intervention period (group 1: *N* = 3; group 2, *N* = 2; group 3, *N* = 2), *N* = 100 completed the (supervised) intervention, 94 completed the full trial (attrition rate of 22%). Further comments: No intervention from 04/20 to 08/20 due to the COVID-19 pandemic. Participation rate Aargau: 10.4% (56 of 540 office workers) and Zurich 8.1% (64 of 793 office workers)

This study was a stepped-wedge cluster randomized controlled trial with each participant completing a control and intervention period [20], i.e., all participants eventually receive the intervention. This is particularly an advantage from an ethical point of view, given that the intervention does more good than harm. Since previous research showed that individual components of our multi-component intervention were effective in reducing neck pain [12, 13, 21], the choice of the design seemed more appropriate compared to other study designs such as the classic cluster RCT. Detailed information can be found in the trial profile (Fig. 1) and the study protocol [18]. Due to the COVID-19 pandemic and the consequent first lockdown in Switzerland, the timing for intervention delivering for the second cluster was delayed by 4 months to August 2020 and by 4 months for cluster 3 to January 2021. Accordingly, the study duration increased from 12 to 16 months. This approach ensured consistency in delivery mode for all participants. Approval was given by the Ethics Committee of the Canton of Zurich, Switzerland (swissethics no. 2019-01678). The CONSORT 2010 Statement extension to cluster randomised trials was used to guide the reporting of the trial [22].

### Participants, Recruitment, and Randomization

Participants had to be office workers aged 18–65 years who worked more than 25 h per week (0.6 full-time equivalent) in a predominantly sitting position, suffered from neck pain or were interested in preventing them, could communicate in German, and gave written informed consent [18]. Participants were excluded if they had a serious health problem that met the European taskforce recommendations [23]: previous trauma or injury to the neck (e.g., neck pain grade 4) [23], specific diagnosed pathology of the neck (e.g., fracture), inflammatory disease of the neck (e.g., spondyloarthropathies), or previous neck surgery [18]. Furthermore, participants who had planned an absence longer than 4 weeks during the intervention and pregnant women were excluded. Participants with known contraindications to performing neck exercises (e.g., on medical advice) were not allowed to participate.

Recruitment took place from October to December 2019 in two medium-sized, governmental-funded Swiss organisations in the cantons of Zurich and Aargau; one was in the higher education sector (Zurich University of Applied Sciences, School of Applied Linguistics and School of Management and Law) and the other in the service sector (Canton Aargau, Department of Civil Engineering, Transport and Environment) [18]. Employees were informed by e-mail, intranet, and during lunch meetings and those interested in participating were asked to register on a website. On a first-come, first-served basis, office workers were then contacted by phone and screened for inclusion and exclusion criteria (AA, Fig. 1).

Participants who worked in the same organisation, on the same floor or in the same room were assigned to the same group (de-identified by AA) to avoid contamination, resulting in a total of 15 groups of 8 participants in each. These 15 groups were then randomly assigned to the intervention cluster (1, 2, and 3) by computer by a senior biostatistician (TV) who was blinded to the identity of the participants. All participants within a cluster changed from the control to the intervention period at the same time and according to the timing of the specific intervention cluster.

### Multi-Component Intervention

The intervention lasted 12 weeks and consisted of a workstation ergonomics intervention, weekly group health-promotion information workshops, and neck exercises [18].

Best practice workstation ergonomics was applied individually by an expert using existing infrastructure (cost-neutral) and an assessment checklist adapted to Swiss guidelines [24]. This 30-min intervention covered topics such as monitor (e.g., position, height), desk (e.g., height), and chair adjustment (e.g., backrest, height), and was carried out once within the first 2 weeks after the commencement of the intervention period. Participants were then instructed to adhere to best practice workstation ergonomics during the rest of the intervention period.

Weekly health-promotion workshops lasted 45 min each and consisted of information and practical activities. Group size was up to 12 participants and the content was discussed in the following order: anatomy of the musculoskeletal system, goal setting, exercise and health, self-efficacy, work stress, digital media and ergonomics, mental health, conflict management, relaxation and sleep, nutrition, resilience and mindfulness, and maintaining motivation. The content was selected on the basis of a previous study [17], in consultation with international experts, and the two organisations involved in this trial. Participants were recommended to attend at least 8 of the 12 workshops.

Participants were instructed to perform neck exercises at a minimum of three times a week for 20 min (1 h per week in total) in a group setting and in a dedicated room at the workplace. One session per week was supervised, and the remaining sessions were self-administered. All participants were given a standard set of 16 exercises targeted to the neck and upper body (Supplementary Information) [17, 18]. The number and selection of exercises within the 20-min sessions and the progression of exercise over the 12 weeks were within the participant’s individual capabilities. At each training session, participants performed warm-up exercises, followed by strength and cool down exercises. The training load for strength exercises was defined at a 10-repetition maximum (10-RM), with two to three sets of 10–15 repetitions. Training intensity was re-assessed during supervised exercises sessions at regular intervals (3, 6, and 9 weeks), progressing from un-resisted to resisted exercises using elastic resistance bands. Between sets, breaks were taken to avoid overexertion. All participants received an app (Physitrack®, London, UK) which could be accessed via smartphone, tablet, or desktop computer. The app displayed a video of each exercise, provided a training reminder and feedback function, and allowed training to be recorded (e.g., number of training sessions).

Interventions were delivered by physiotherapists, movement scientists, and/or Master of Psychology students. All were trained for at least 4 h before intervention commencement. Participants could report the time spent for interventions as working time, except for the unsupervised neck exercise sessions only. Office workers who had already completed the intervention period were advised by the research team to continue training on an unsupervised basis. Due to the COVID-19 pandemic, the intervention was delivered from March 2020 onwards in a hybrid format (participation on-site or via video teleconference). It was not mandatory that all participants in the same cluster received the intervention on the same day of the week, so the group size could be larger than eight.

### Outcomes

The five measurement time points (baseline, follow-up 1–4) at 4-month intervals are shown in the trial profile (Fig. 1). Regardless of whether the participant was in the intervention or control period, measurements were made at the same time point for all participants. All data were obtained using online questionnaires, each taking about 30–45 min to complete, and were hosted by the tool UNIPARK© (Berlin, Germany). Participants could report the time spent for completing the questionnaires as working time.

#### Primary Outcome

The primary outcome of neck pain-related work productivity loss was expressed as a percentage of weekly working time. It was quantified with the Work Productivity and Activity Impairment Questionnaire for Specific Health Problem (WPAI, German version) [25], which includes the following questions with a recall frame of 1 week: Q1 = currently employed; Q2 = hours missed due to neck pain; Q3 = hours missed due to other reasons (e.g., vacation); Q4 = hours actually worked; Q5 = degree to which neck pain affected work productivity (on a Numeric Rating Scale NRS ranging from 0 = not at all to 10 = maximum) [25]. Neck pain-related percentages of absenteeism (Q2/(Q2 + Q4) and presenteeism ((1-absenteeism) * Q5/10)) were calculated according to the scoring rules of the developers and summed to obtain the neck pain-related work productivity loss [25]. The monetary value of neck pain-related work productivity loss (in CHF) was calculated [25], which is described in detail in statistical analysis section.

#### Additional Variables

Other information collected included: employer (Zurich, Aargau), workload percentage (< 80%, 80–89%, 90–99%, 100%; 100% corresponds to 42 h per week), work role (with or without a leadership responsibilities), education level (tertiary level education, non-tertiary level education), average weekly earnings (in CHF), civil status (married, not married but in a relationship, not married and not in a relationship), nationality (Swiss, non-Swiss), intensity of the neck pain (Numerical Rating Scale (NRS) from 0 = no pain to 10 = maximum pain), gender (male, female), first onset of neck pain (in months), and age. Work-related stress conditions were assessed using the Job-Stress-Index (JSI). The JSI is based on validated questionnaires and represents the ratio of work-related resources (e.g., holistic work tasks) to stressors (e.g., time pressure) [26]. It ranges from 0 to 100, with a value below 45.879 representing a favourable range (resources > stressors), a sensitive range of 45.880–54.122 (resources = stressors), and a critical range above 54.123 (resources < stressors) [26].

### Sample Size Calculation

For sample size calculation, a baseline work productivity of 90% and an intervention-related work productivity increase of 5% were assumed [17]. Type I Error was set at alpha = 0.05 and Type II Error at beta = 20% (power = 80%). We decided for the scenario of 12 groups with six participants each, but due to the attrition rate of nearly 20% of a previous Australian study [17] we increased the number of groups and subjects per group by 20% each [18, 27]. Thus, we enrolled 120 participants over 15 groups for four measurement time points (480 observations). As described in the study design section, the study duration increased from 12 to 16 months due to the COVID-19 pandemic. Therefore, an additional (fifth) measurement time point was added (follow-up 4) thus increasing the number of observations to 600.

### Statistical Analysis

Descriptive statistics with mean, median, standard deviation, maximum, and minimum value were used to characterize participants. Where variables were nominal or ordinal (e.g., gender), relative and absolute frequencies were reported.

For the primary outcome, a generalized linear mixed-effects model of the Gaussian family with log-link was fitted to the data to estimate the change in neck pain-related work productivity loss [28]. The model included a random intercept term to account for repeated measurements on the same cohort of participants as well as fixed effects for intervention cluster (cluster 1, 2, or 3), treatment (intervention, control), and time (measurement time point; baseline, follow-up 1, follow-up 2, follow-up 3, follow-up 4) [28]. The latter provided indication of whether conditions during the COVID-19 pandemic were the same for all study participants. Due to the study design, sample size calculation and statistical analysis plan (i.e., limited degrees of freedom), no further control for a confounding effect of the COVID-19 pandemic was possible. Furthermore, the model included fixed effects for age, gender, education level, civil status, nationality, employer, workload percentage, work role, and work stress conditions (JSI) to adjust for potential confounding effects. Average marginal effects were derived from the model in order to estimate changes in work productivity. The weekly monetary value of neck pain-related work productivity loss was derived by multiplying the weekly earnings by the weekly adjusted productivity loss (based on the model presented above) for both treatment groups (intervention, control), and the costs saved were the difference thereof.

The statistical analyses were performed using Stata® Version 15.1 (StataCorp, College Station, Texas, USA) and R® (Boston, USA) statistical software. Significance level was set at alpha = 0.05. We reported all model estimates with corresponding 95% confidence intervals (95% CI). Data analysts were blinded to the identity and group allocation of the participants. The data were analysed on an intention-to-treat basis. The study was registered on ClinicalTrials.gov (NCT04169646, https://clinicaltrials.gov/ct2/show/NCT04169646, study completed).

## Results

Participants were recruited between Oct 28, 2019, and Dec 20, 2019. Data from 120 participants, amounting to 517 observations with an average of 4.3 observations per participant, were included in the analysis. A total of 21 observations were missing. We experienced a total of 26 dropouts (male 9, female 17; attrition rate: 22%; Fig. 1), with 13 office workers dropping out before the start of the intervention (= 31 observations), 7 during the intervention (= 18 observations), and 6 after the intervention (= 13 observations).

Participants’ characteristics are shown in Table 1. The mean age was 43.7 years (SD 9.8 years) and the distribution by employer was balanced (Zurich: 53.3%, *N* = 64). The majority of participants were female (*N* = 86, 71.7%), Swiss (*N* = 95, 79.2%), in a relationship (married: *N* = 48, 40%; not married: *N* = 53, 44.2%), and had a tertiary level education (*N* = 89, 74.2%). In terms of workload, most participants worked full-time (*N* = 67, 55.8%), had no leadership responsibilities (*N* = 76, 63.3%), and the average monthly earnings was CHF 7679 (SD 2818).

**Table 1:** Participant characteristics at baseline

	Baseline (*N* = 120)
*Workload percentage*	
< 80 (%)	25 (20.8%)
80—89 (%)	28 (23.3%)
90—99 (%)	19 (15.8%)
100 (%)	48 (40.0%)
*Job-Stress-Index [0–100]*	
Mean (SD)	47.6 (5.0)
Median (IQR)	46.8 (6.2)
*Job-Stress-Index [categories]*	
Favourable range (JSI below 45.879; resources > stressors; %)	50 (41.7)
Sensitive range (JSI between 45.880 and 54.122; resources = stressors, %)	54 (45.0)
Critical range (JSI above 54.123; resources < stressors; %)	16 (13.3)
*Neck pain-related work productivity loss [% of working time]*	
Mean (SD)	12.0 (19.4)
Median (IQR)	0 (12.5)
*Neck pain-related presenteeism at work [% of working time]*	
Mean (SD)	10.8 (16.9)
Median (IQR)	0 (10.0)
*Neck pain-related absenteeism at work [% of working time]*	
Mean (SD)	1.2 (9.2)
Median (IQR)	0 (0)

*IQR* = interquartile range; *SD* = standard deviation

On average, the first onset of neck pain was 42.9 months before baseline measurement (range from 0 to 368 months), and 45% of participants (*N* = 54) reported suffering from chronic neck pain (3 months or longer). Approximately 88% of participants (*N* = 106) suffered from neck pain at least at one measurement point, with 95 (79.2%) participants reporting neck pain at baseline with a mean intensity of NRS 3.0 (SD 1.8, Median 2.0, Min 0.0, Max. 9.0, IQR 2.0). Participants with neck pain at baseline (*N* = 95) reported a higher neck pain-related work productivity loss (14.9%, with 1.48% for absenteeism and 13.5% for presenteeism) than participants without neck pain (*N* = 25, 0.8%, with 0% for absenteeism and 0.8% for presenteeism).

Out of the 107 participants who were in the intervention period, 27.1% (*N* = 29) were adherent to the neck exercises (mean = 31.2 training sessions, range from 0 to 83 training sessions), 61.7% (*N* = 66) were adherent to health-promotion information (mean = 8.2 group workshop attendances, range from 0 to 12 group workshop attendances), and 97.2% (*N* = 104) were adherent to workplace ergonomics (Supplementary Information).

Adjusted for all confounders, the intervention was negatively associated with neck pain-related work productivity loss (b = −0.27; 95% CI ranging from −0.54 to −0.001) yielding an average marginal treatment effect of −2.8 percentage points in the observed population. For instance, in a simplified example, an office worker working 42 h per week would report a neck pain-related work productivity loss of 10% (4.2 h per week) before the intervention. After the intervention, the same office worker would report a neck-pain-related work productivity loss of 7.2% (3 h per week), assuming all other confounders remain constant as observed. For measurement time points, intervention clusters and the two different organisations, no association with neck pain-related work productivity loss was found (i.e., no confounding effect). With respect to the covariates, men as compared to women showed less productivity loss (−0.58; 95% CI ranging from −1.12 to −0.03). Similarly, productivity loss was negatively associated with older age (−0.05; 95% CI ranging from −0.08 to −0.27), and tertiary education (−0.54; 95% CI ranging from −1.12 to −0.03). Higher productivity loss was associated with increased work stress conditions (JSI, 0.03; 95% CI ranging from 0.005 to 0.05), not being married (in relationship: 0.79; 95% CI ranging from 0.22 to 1.35; without partner: 0.99; 95% CI ranging from 0.28 to 1.70), and not having leadership responsibilities (work role; 0.85; 95% CI ranging from 0.33 to 1.37). No association was found for nationality and workload percentage with neck pain-related work productivity. The adjusted model is presented in Table 2; the unadjusted model can be found in the Supplementary Information.

**Table 2:** Neck pain-related work productivity loss (%), adjusted model with 517 observations

	Coefficient	95% confidence interval	*p*-value
*Treatment, intervention (Ref = control)*	−0.27	From −0.54 to −0.001	0.049
*Measurement time point (Ref = Baseline, January 2020)*			
Follow-up 1 (April 2020)	−0.01	From −0.26 to 0.23	0.93
Follow-up 2 (August 2020)	0.17	From −0.05 to 0.40	0.13
Follow-up 3 (November 2020)	0.02	From −0.26 to 0.31	0.86
Follow-up 4 (April 2021)	0.16	From −0.20 to 0.52	0.38
*Intervention cluster (Ref = Cluster 3, January to April 2021)*			
Cluster 1 (January to April 2020)	−0.54	From −1.08 to 0.01	0.053
Cluster 2 (August to November 2020)	−0.39	From −0.90 to 0.12	0.14
*Age*	−0.05	From −0.08 to −0.03	< 0.001
*Gender, male (Ref = female)*	−0.58	From −1.12 to −0.03	0.04
*Education, tertiary (Ref = non-tertiary level)*	−0.54	From −1.12 to 0.03	0.07
*Civil Status (Ref = married)*			
Not married, in a relationship	0.79	From 0.22 to 1.35	0.01
Not married, not in a relationship	0.99	From 0.28 to 1.70	0.01
*Nationality, Non-Swiss (Ref = Swiss)*	0.37	From −0.13 to 0.88	0.15
*Employer, Aargau (Ref = Zurich)*	0.03	From −0.50 to 0.55	0.93
*Workload percentage (Ref = 100%)*			
90–99%	−0.37	From −1.03 to 0.29	0.28
80–89%	−0.15	From −0.71 to 0.41	0.59
< 80%	0.49	From −0.15 to 1.13	0.13
*Work role, with leadership responsibilities (Ref: without leadership responsibilities)*	0.85	From 0.33 to 1.37	0.001
*Job-Stress-Index*	0.03	From 0.005 to 0.05	0.02
*Model Constant*	1.71	From 0.88 to 2.54	< 0.001
*Random Intercept Variance (participants)*	0.69	From 0.44 to 1.10	
*Residual Variance*	150.52	From 132.06 to 171.55	

The predicted monetary value of neck pain-related work productivity loss was CHF 183.90 in the control group (SD 246.70) and CHF 156.50 in the intervention group (SD 204.70) which corresponds to weekly saved costs of CHF 27.40 per participant in the intervention group.

During the control period, one adverse event occurred after a physical examination of the neck (hearing loss and tinnitus) resulting in a medical consultation. Physical examination of the neck, e.g., neck flexor strength, was a secondary outcome of this study and will be reported in a different paper [18].

## Discussion

### Summary of Findings

About 80% of our sample of Swiss office workers reported mild to moderate neck pain (average NRS 3.0/10) at baseline and neck pain-related work productivity loss was 12% of working time at baseline (combination of absenteeism and presenteeism). We found an effect of our multi-component intervention on neck pain-related work productivity by −2.8 percentage points and weekly saved costs of CHF 27.40 per participant. In addition, a negative effect for the covariates of male gender, older age, and tertiary education level on the loss of work productivity was found. Increased work stress conditions (JSI), not being married, and not having leadership responsibilities were positively associated with neck pain-related work productivity loss. Our hypothesis that the intervention could reduce the economic burden of neck pain in office workers was confirmed by these findings.

### Comparison with Literature

Overall, our findings are consistent with existing literature, although the study conditions changed due to the COVID-19 pandemic. Justesen and colleagues [29] investigated the effect of a 12-month individual physical exercise programme combined with moderate-intensity activity and found evidence for a reduction in absenteeism, presenteeism, and productivity loss at work, but only for office workers with high adherence to the intervention. The Australian study of Pereira and colleagues [17] compared two 12-week intervention programmes, with participants who attended a workplace ergonomics intervention and neck exercise programme showing a lower health-related work productivity loss at 12-month follow-up than those who attended a workplace ergonomics and health promotion programme. Their monthly saved health-related work productivity costs amounted to CHF 186 ($ 276) at 1-year follow-up [17], but it must be clearly highlighted that both intervention groups had higher health-related work productivity losses compared to baseline. Nonetheless, their value of CHF 186 at 1-year follow up is very similar to the value of saved costs from our study of about CHF 27.40 CHF per participant per week, considering that we only recorded the productivity losses at work due to neck pain.

### Interpretation of Treatment Effect

#### Effect Size

An absolute change in WPAI score of 7–20% is reported as a minimal clinically important difference [30, 31], but the associated studies did not include patients with musculoskeletal conditions. Using this reference value, we classify our treatment effect as small. Nevertheless, there are several things to consider when interpreting our values. Firstly, we expected a relatively high treatment effect, i.e., a reduction in the observed work productivity losses by 5 percentage points (reduction from 10% productivity loss to 5%; in relative terms: 50%) [17]. Our observed (predicted) treatment effect of −2.8 percentage points was lower than the expected value, though still equivalent to a relative reduction of 23.5% in work productivity losses due to neck pain compared to the baseline productivity losses of 12%. With regard to the sample size calculation, this discrepancy between the expected and observed treatment effect reduces the power of our findings. Secondly, the burden of neck pain was comparatively low in our sample [32]: 80% of participants reported mild to moderate neck pain and 20% had no neck pain at baseline, which may indicate a floor effect and may have diluted the observed treatment effect. Possible reasons include the time between recruitment and baseline measurement of several weeks. This in combination with an intermittent occurrence of neck pain and a recall period of 4 weeks may have resulted in fewer recordings of neck pain. A regression to the mean, in contrast, was controlled by using multiple measurement time points. To summarise the first and second statement: Our intended goal of a relative reduction of 50% in neck pain-related work productivity loss seems quite ambitious in a sample with low levels of neck pain. Nevertheless, we were able to demonstrate a statistically significant, albeit small, treatment effect of our multicomponent intervention. Thirdly, a small treatment effect in a study—which may not be clinically relevant to the individual—may still imply a larger effect at the population or worker level due to a shift in the population curve. In other words, a small treatment effect relative to the large number of people affected by neck pain may represent an important public health impact.

#### Risk of Overtreatment

There is a risk of overtreatment due to our study design. All individuals received the same intervention, regardless of their level of pain, and there was no individual matching to the intervention, making the intervention time-consuming to deliver and participate in. This should not be underestimated, especially when considering a similarly high treatment effect as in other studies, but a comparatively larger time investment for the participants.

#### Representativeness of the Sample and Results

For an international comparison, three main aspects must be considered when interpreting our results from Switzerland. First, our sample was representative of office workers in terms of age [33], but not in terms of education level [34]. Current literature shows a negative association between work productivity losses and educational level [35], potentially due to better health literacy. However, since we measured the productivity loss as a percentage, it is unclear whether and what impact the different education levels had on the treatment effect. Second, the reported monthly earnings are without tax deduction (which would correspond to a monthly reduction in earnings of roughly 20% if taxes are included), which makes them seem very high. Third, Switzerland has a different social and medical insurance system than other countries. Workers only need a medical certificate for an absence of more than three days due to illness, and their wages continue to be paid during this time. This could lower the barrier to absenteeism, but there are special rules and wage deductions for long-term absences.

#### COVID-19 Pandemic

It should not be neglected that our study started shortly before the COVID-19 pandemic restrictions became effective in Switzerland. There was a national requirement to work from home during our 16-month study period (recommendation: 58 weeks, requirement: 23 weeks), which changed not only the work environment, but also to some extent the working hours, work tasks, and private commitments. As shown in the study profile, it was therefore decided not to move any cluster into the intervention period in April 2020. At that point, it was assumed that the COVID 19 pandemic would end in August 2020, and if not, this would provide sufficient time to prepare for the switch to the hybrid setting. This short-term interruption of the study thus affected all participants equally, regardless of whether they were in the intervention or the control period. The main consequence for the participants was the fact that an additional measurement point had to be included (follow-up 4).

Still, one could argue the COVID-19 pandemic may have had an impact on our results in terms of dose–response (e.g., time of sedentary desk work, intensity of exercises) and attrition rate, which are both described as highly relevant predictors on treatment outcomes [36]. For example, half of the dropouts had already discontinued participation before the start of their intervention period (*N* = 13). However, the fact that the measurement time point was not statistically significant is an indicator that all participants had the same conditions during the study (i.e., no substantial change, [37]). This is also confirmed by our research group that there was neither evidence of a decrease in physical activity [38], nor of a change in neck pain intensity or disability [39], nor of a change in work stress conditions [40] in our sample of Swiss office workers during the COVID-19 pandemic (follow-up 1 measurement in April 2020, working from home) compared to the situation before the COVID-19 pandemic (baseline measurement in January 2020, working at the office). We were only able to show that workplace ergonomics was rated to be worse at home than in the office [41]. Nevertheless, it remains unclear to what extent the results can be transferred to everyday office life without COVID-19.

### Interpretation of Covariates

With regard to the covariates, the loss of neck pain-related work productivity was found to be lower in older participants, which could be explained by the more consolidated personality traits, better stress coping strategies, overall greater (work) experience, and healthy worker effect. This proposition is supported by our findings, showing that office workers who are exposed to increased work-related stressors [42, 43], who have leadership responsibilities, or who are not being married also tend to have higher productivity losses at work. In addition, our findings confirm that the work productivity loss due to neck pain is significantly higher in women than in men [44].

### Study Design

The stepped-wedge cluster randomized study design has been criticized by Kotz and colleagues [45] on various grounds. According to Kotz and colleagues [45], the most important disadvantage is that an intervention is implemented in all clusters, whereas it has not yet proven to be effective. However, in our case several components of our intervention already proved to be effective in other studies [12, 13, 17, 21, 29]. Hence, a stepped-wedge design is superior both scientifically (more data) and ethically as has been pointed out by Mdege and colleagues [46]. Moreover, most of the points raised by Kotz and colleagues [45] also apply to other study designs [46].

### Strengths

This study has several strengths. First, we included employees with and without neck pain, which is why our results are representative for the treatment and prevention of neck pain-related work productivity losses in office workers in general. In this way, the fluctuating nature of neck pain in office workers can be addressed more appropriately. Second, the study design minimized contamination between groups, but still allowed all participants to receive the intervention. Third, the primary outcome allowed differentiation between neck pain-related absenteeism and presenteeism at work, and not only sick leave and productivity as in previous studies [11, 36]. Fourth, current recommendations for the successful implementation of such a programme were applied: medium to large companies were recruited, the intervention was carried out in the workplace and during working hours, it included training programmes and information material, and was supervised [47, 48]. Fifth, the components of the intervention were selected according to the current best available evidence, which was intended to reduce neck pain and work productivity losses in office workers. Sixth, the intervention could be continued in a hybrid setting despite the COVID-19 pandemic. And finally, the intervention could be implemented or replicated with little effort as all content is available digitally: the exercises as videos on an app, the workshops as podcasts, and the workplace ergonomics in the form of a checklist.

### Limitations

The high level of education, average earnings, employment by a local government, and gender distribution may have affected the transferability and comparability of our findings to other jurisdictions and samples of office workers. There may have been a selection bias as only those who had sufficient resources (e.g., time) and with mild to moderate burden of disease registered for the study participation. For our primary outcome, self-reported questionnaires were used, which are controversial because of their accuracy and potential social desirability bias. Some follow-up measurements were conducted close to holidays, so participants may not have reported neck pain or productivity losses due to vacation. Medication use or other forms of intervention (e.g., physiotherapy) were not recorded, which may have affected neck pain. Furthermore, making up for missed work hours (e.g., working overtime in another week) was not considered in the questionnaire of the primary outcome, which could lead to an overestimation of neck pain-related work productivity losses. Another limitation is the COVID-19 pandemic with the change in working conditions and the switch to a hybrid setting of our intervention, which might have biased the adherence to the intervention and the dose–response relationship, e.g., for deskwork or neck exercises. This, in turn, could lead to an underestimation of the treatment effect.

### Further Research

Based on our results, a cost-benefit and cost-utility analysis should be conducted to obtain a better understanding of the true health economic impact of our multi-component intervention. In addition, the effect of the COVID-19 pandemic (e.g., working from home versus working at the office, [37]) and the season (e.g., flu season in January versus August) on neck pain-related work productivity loss should be investigated using longitudinal data [49]. Future studies should compare different intervention durations (i.e., dose–response, e.g., 8 and 12 weeks), control for the intake of pain relief medication and physical activity level, allow the selection of health promotion workshop content at a participant level according to their needs, investigate the sustainability of the effect (e.g., need for boosters), and include office workers with at minimum mild neck pain.

## Conclusion

As neck pain has an impact on the individual and society and the nature of work is increasingly moving towards prolonged computer work, the burden and treatment of neck pain becomes more important. Our findings provide evidence on strategies employers and policy makers can use to improve health-related productivity by reducing absenteeism and presenteeism among office workers.

## Supplementary Information

Below is the link to the electronic supplementary material.

Supplementary file1 (PDF 778 KB)

## Data Availability

The data that support the findings of this study are available from the corresponding author upon request.
