# Supplementary material for: A Multi-component Intervention (NEXpro) Reduces Neck Pain-Related Work Productivity Loss: A Randomized Controlled Trial Among Swiss Office Workers
Source: J Occup Rehabil. 2022 Sep 27;33(2):288–300. doi: 10.1007/s10926-022-10069-0 (PMC9514678; doi:10.1007/s10926-022-10069-0)
Supplement: Supplementary file 1 — Supplementary file1 (PDF 778 KB) [file 10926_2022_10069_MOESM1_ESM.pdf]

Supplementary Information

Article title: A multi-component intervention (NEXpro) reduces neck pain-related work productivity loss: A randomized controlled trial among Swiss office workers

Journal name: Journal of Occupational Rehabilitation

Author names: Aegerter AM, Deforth M, Volken T, Johnston V, Luomajoki H, Dressel H, Dratva J, Ernst MJ, Distler O, Brunner B, Sjøgaard G, Melloh M and Elfering A on behalf of the NEXpro collaboration group

Corresponding author: Andrea M Aegerter, ZHAW Zurich University of Applied Sciences, School of Health Sciences, Institute of Public Health, Katharina Sulzer-Platz 9, 8400 Winterthur, Switzerland; andrea.aegerter@zhaw.ch; +41 58 934 67 91

Table S1 Neck pain-related work productivity loss (%), unadjusted model with 517 observations

	**Coefficient**	**95 % confidence interval**	**p-value**
**Treatment, intervention** (Ref = control)	-0.19	from -0.45 to 0.07	0.16
**Measurement time point** (Ref = Baseline, January 2020)			
Follow-up 1 (April 2020)	-0.08	from -0.32 to 0.17	0.54
Follow-up 2 (August 2020)	-0.08	from -0.14 to 0.30	0.49
Follow-up 3 (November 2020)	-0.08	from -0.35 to 0.20	0.60
Follow-up 4 (April 2021)	-0.001	from -0.36 to 0.36	0.99
**Intervention cluster** (Ref = Cluster 3, January to April 2021)			
Cluster 1 (January to April 2020)	-0.55	from -1.15 to 0.04	0.07
Cluster 2 (August to November 2020)	-0.20	from -0.73 to 0.34	0.47
Model Constant	2.00	from 1.54 to 2.47	< 0.001
Random Intercept Variance (participants)	1.20	from 0.78 to 1.84	
Residual Variance	151.65	from 132.85 to 173.11	

Table S2 Adherence to intervention

	**(N=107)**
**Number of neck exercise training sessions, over 12 weeks**	
Mean (SD)	31.2 (13.3)
Median (IQR)	31.0 (10.5)
**Adherence to neck exercises** [1]	
Adherent (%)	29 (27.1)
Exceeded the recommendation (> 3 training sessions a week)	25 (23.4)
Met the recommendation (= 3 training sessions a week)	4 (3.7)
Non-adherent (%)	78 (72.9)
Slightly below the recommendation (2 to < 3 training sessions a week)	56 (52.3)
Clearly the recommendation (1 to < 2 training sessions a week)	13 (12.2)
Very clearly below the recommendation (< 1 training session a week)	9 (8.4)
**Number of health-promotion information group workshop attendances, over 12 weeks**	
Mean (SD)	8.2 (2.8)
Median (IQR)	8.0 (3.0)
**Adherence to health-promotion information** [2]	
Adherent (%)	66 (61.7)
Exceeded the recommendation (> 8 workshop attendances)	52 (48.6)
Met the recommendation (= 8 workshop attendances)	14 (13.1)
Non-adherent (%)	41 (38.3)
Slightly below the recommendation (5 to 7 workshop attendances)	32 (29.9)
Clearly below the recommendation (< 5 workshop attendances)	9 (8.4)
**Adherence to workplace ergonomics** [3]	
Adherent (%)	104 (97.2)
Non-adherent (%)	3 (2.8)

Key: IQR = interquartile range; SD = standard deviation

[1] Participants were classified as being “adherent to neck exercises” if they have followed our recommendation to exercise 3 times a week over a period of 12 weeks (≥ 36 training sessions over 12 weeks = adherent, < 36 training sessions over 12 weeks = non adherent).

[2] Participants were classified as being “adherent to health-promotion information” if they have followed our recommendation to attend at minimum 8 / 12 workshops (≥ 8 workshop attendances = adherent, < 8 workshop attendances = non adherent).

[3] Participants were classified as being “adherent to workplace ergonomics” if they attended the 30-minute workplace ergonomics intervention.

Figure S1: Set of 16 neck exercises

Bilateral scapular raise

3x10 repetitions

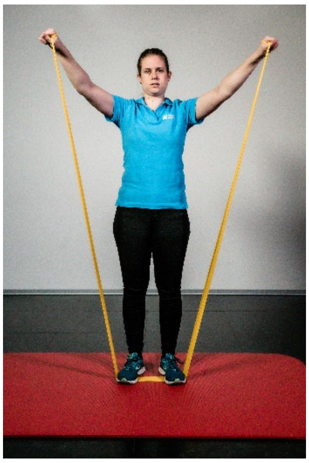

Bilateral shoulder shrugs

3x10 repetitions

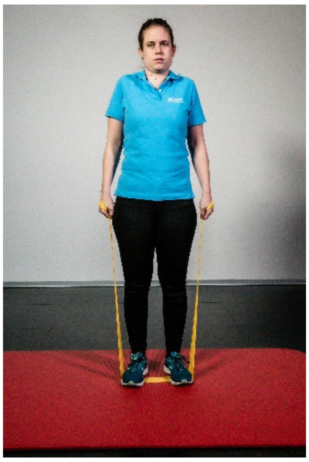

Row

3x10 repetitions

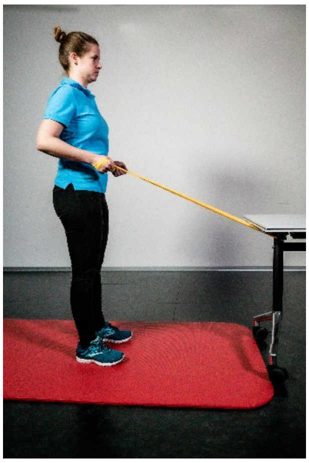

Bilateral shoulder extension

3x10 repetitions

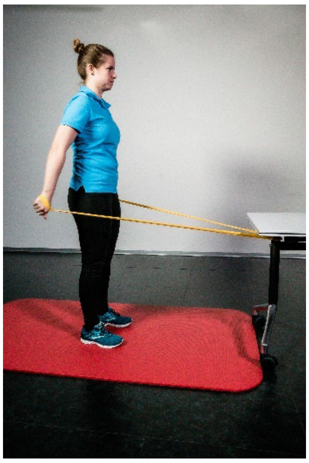

Bilateral shoulder external rotation, *3x10 repetitions*

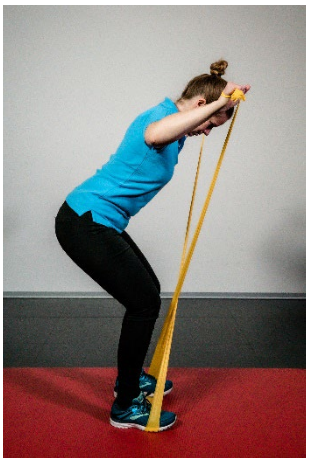

Bench dips

3x10 repetitions

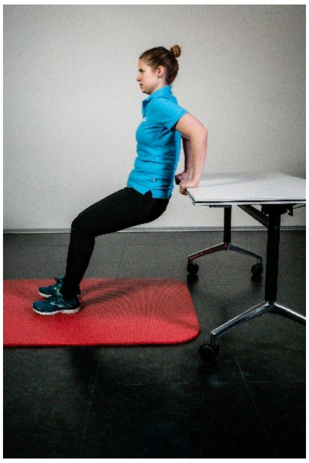

Seated side stretch

3x20 seconds, each side

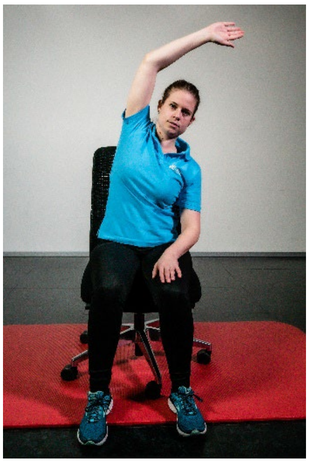

Self-massage

3x20 seconds

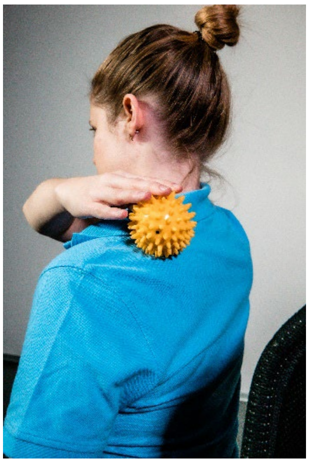

**Isometric neck flexion**<br>*5x5 seconds*<br>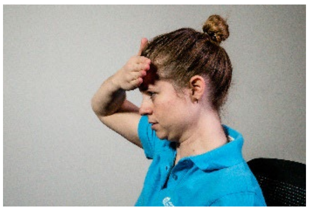<br>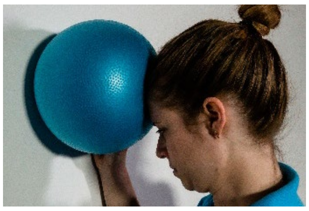	**Isometric neck extension**<br>*5x5 seconds*<br>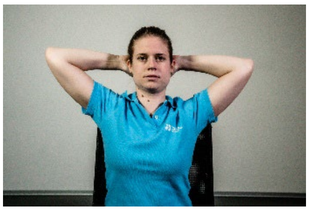<br>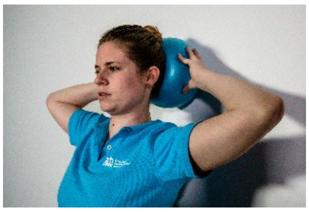	**Isometric neck rotation**<br>*5x5 seconds, each side*<br>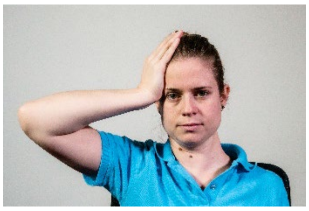<br>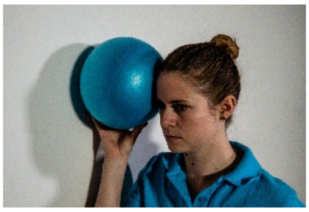
**Push-ups**<br>*3x10 repetitions*<br>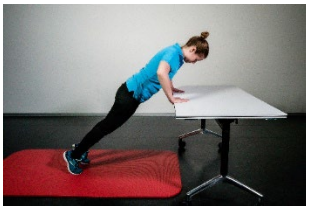	**Bilateral shoulder circling**<br>*1x20 seconds*<br>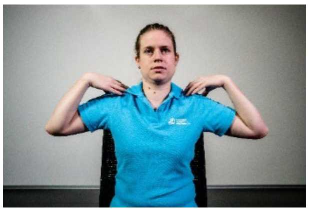	**Upper body rotation**<br>*1x20 seconds, each side*<br>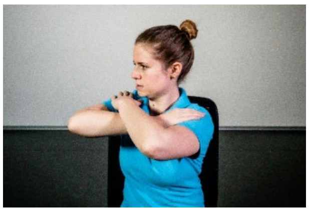
**Stretch of neck extensor**<br>*3x20 seconds*<br>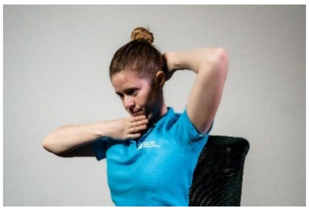	**Lateral neck stretch**<br>*3x20 seconds, each side*<br>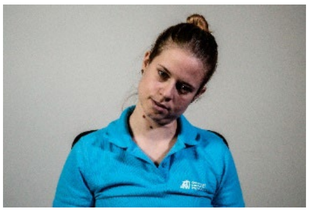	

Key: They not only strengthen the neck, but also the shoulders, arms and back.
